# Predicting health behaviors during the COVID-19 pandemic: A longitudinal study

**DOI:** 10.1371/journal.pone.0299868

**Published:** 2024-03-15

**Authors:** Robin Wollast, Mathias Schmitz, Alix Bigot, Marie Brisbois, Olivier Luminet

**Affiliations:** 1 Research Institute for Psychological Sciences, UCLouvain, Ottignies-Louvain-la-Neuve, Belgium; 2 Faculty of Psychological Sciences and Education, Université libre de Bruxelles, Bruxelles, Belgium; 3 Fund for Scientific Research (FRS-FNRS), Brussels, Belgium; Federal University of Paraiba, BRAZIL

## Abstract

We investigated the social, emotional, and cognitive predictors of adherence to four health behaviors (handwashing, mask wearing, social contact limitations, and physical distancing) during one critical phase of the COVID-19 pandemic. We collected data (*N* = 5803, mean age = 53; 57% women) in Belgium at five time points between April and July 2021, a time during which infections evolved from high (third wave of the pandemic) to low numbers of COVID-19 cases. The results show that the social, emotional, and cognitive predictors achieved high levels of explained variance (*R*^*2*^ > .60). In particular, the central components of behavioral change (attitudes, intentions, control, habits, norms, and risk) were the strongest and most consistent predictors of health behaviors over time. Likewise, autonomous motivation and empathetic emotions (e.g., attentive, compassionate) had a positive impact on health behavior adherence, whereas it was the opposite for lively emotions (e.g., active, enthusiastic). These results offer policymakers actionable insights into the most potent and stable factors associated with health behaviors, equipping them with effective strategies to curtail the spread of future infectious diseases.

## Introduction

Since early 2020, the world has grappled with an ongoing pandemic caused by the spread of COVID-19. In this critical context, health behaviors have emerged as pivotal measures to curb the virus’s transmission, garnering support from governments and various health institutions. As a result, the identification of socio-psychological factors that either facilitate or hinder people’s adherence to these sanitary measures became vital. Understanding how people adhere to health behaviors not only aids in addressing the current health crisis, but also equips policymakers to better manage future health emergencies.

One notable limitation of many previous studies on health behaviors lies in the fact that they primarily relied on one-time-point designs, which are considered a limited set of predictive factors, and on single or very few items to measure health behaviors. To overcome these limitations, the present research adopted a longitudinal design from April to July 2021, capturing data during the third pandemic wave in Belgium. In addition, we assessed the predictive power and stability of a wide array of psychological factors over time. Finally, we examined four crucial health behaviors—handwashing, mask wearing, social contact limitations, and physical distancing—using multiple items to account for the varied contexts in which these behaviors occurred.

### Health behaviors

Since the onset of the outbreak, the World Health Organization [1] and governments worldwide have advocated for adopting a set of preventive health measures aimed at curbing the spread of the virus. In our current research, we focus on four of these crucial preventive measures: handwashing, mask wearing, social contact limitations, and physical distancing. These health behaviors have demonstrated their effectiveness in reducing the transmission of the virus [2].

Despite their efficiency in flattening the epidemic curve, these behaviors significantly vary in nature [3]. Handwashing is a conventional practice familiar to a vast portion of the population, and its application is easy. Moreover, it aligns with societal norms and garners widespread acceptance. Importantly, it does not encroach upon any fundamental human needs. Conversely, the other three behaviors, often termed avoidant behaviors, run contrary to the inherent social nature of humans. Furthermore, they were entirely new to the majority of the population and did not conform to established social norms. As expected, they pose a much greater challenge to enact, potentially resulting in adverse psychological consequences, notably anxiety and depression, because they push against deep human’s instinct for togetherness [4, 5].

In this context, Wollast and colleagues [5] discovered that participants reporting the highest levels of adherence to social contact limitations and physical distancing over time also experienced increased feelings of loneliness and decreased life satisfaction, with a marginal effect observed for mask wearing. However, this phenomenon was not observed among participants who showed high levels of handwashing. This suggests that, although these health behaviors are interconnected, their impact on mental health substantially varies.

### Predictors of health behaviors

The current study focused on several key predictors drawn from the health behavior literature, including socio-demographics, the central components of socio-cognitive models of behavior change (CCCM), emotions, motivations, and COVID-19-related measures. Below, we provide a concise summary of the pertinent findings for each category of predictors.

Regarding socio-demographics, it is noteworthy that, in general, women tend to demonstrate greater adherence to health behaviors [6–8]. This gender disparity may partially arise from the perception of a higher risk of COVID-19 infection among women compared with men [9, 10]. In this context, researchers found that women reported wearing a mask more often than men because masks may have been viewed as a sign of fragility or weakness [11, 12]. Specifically, men were more likely to have perceived sanitary rules as infringing on their independence, indicating that men and women have differing perceptions and adoptions of health behaviors [13, 14]. A similar trend is often observed concerning age: adherence tends to increase with advancing age [5, 7, 11, 15, 16]. However, findings concerning education level are less straightforward. Although social contact limitations are generally more adhered to by individuals with higher levels of education, the results for handwashing and mask wearing show greater ambiguity [6, 7, 15, 17–19].

Similarly, research has demonstrated associations between health behavior adherence and psychological factors [20–24]. Therefore, we considered the central components from two of the most prominent socio-cognitive models of behavior change: the theory of planned behavior (TPB [25]) and health action process approach (HAPA [26]). Both have been successfully used as robust frameworks to explain the underlying mechanisms of health behaviors adoption during the COVID-19 pandemic [3, 27]. These theories posit that health behaviors can be predicted by the *intentions* to perform the behavior, *attitudes* toward the behavior (i.e., whether people think it is useful, important, or desirable), the *control* that people perceive they have over their actions, and the *social norms* (i.e., descriptive norms: what people do; prescriptive norms: what should be done; and moral norms: what is “right” to do). In this context, studies have shown that these models are robust in explaining health behavior adherence across countries [28].

These models have been extended and include additional constructs, such as *habits* (automatic enactments of a behavior developed through its repeated performance) and *perceived risk of infection* (personal perceived probability of getting infected). Many studies have shown that these factors were strongly and positively associated with health behaviors adoption in various contexts, including the pandemic [28–33].

One criticism leveled against socio-cognitive models of behavior change is their tendency to prioritize cognitive components at the expense of affective ones, even though it is worth noting that attitudes do encompass some affective dimensions [34]. Previous investigations that have delved into the influence of emotional states on predicting health behaviors have primarily focused on the impact of stress, with limited exploration of specific emotional states [35]. Moreover, when studies have explored discrete emotional states, the emphasis has often been on fear alone [36]. Although fear plays a substantial role as an effective response during pandemics [37], it is imperative to consider a broader spectrum of emotional states. Recent studies conducted during the early stages of the pandemic have underscored the significance of these affective predictors [7, 19]. In particular, the findings have indicated that heightened attentiveness, anger, or anxiety fostered handwashing, while increased anger or decreased happiness encouraged limitations of social contact.

Individuals adhere to these measures for a variety of reasons. One way to account for the underlying motivations is through the lens of self-determination theory (SDT [38, 39]), which has seen limited use within the context of a pandemic. In contrast to the socio-cognitive models mentioned earlier, which have primarily relied on quantitative perspectives (e.g., assessing the degree of personal control), SDT adopts qualitative distinctions among different types of motivation. Researchers have distinguished between autonomous and controlled motivation [40]. Autonomous motivation signifies an intrinsic understanding of the meaning and value of a behavior, while controlled motivation stems from external pressures, such as government mandates or the desire to avoid penalties, to perform the behavior. In the context of health behaviors, individuals may adhere to the measures either because they genuinely comprehend and identify with the guidelines (i.e., autonomous motivation) or because they feel coerced by external forces or penalties (i.e., controlled motivation). Scholars [9] demonstrated that autonomous motivation serves as a robust predictor of sustained compliance with COVID-19 measures, while controlled motivation exhibits no enduring predictive value (see also [32], regarding vaccination intention and uptake).

Finally, in terms of COVID-19 risk-related demographic variables, we examined whether people had been infected in the past and whether they were vaccinated. These factors have been shown to be strong predictors of adherence to health measures [9, 22, 30, 32]. In sum, there is a plethora of predictors that have been associated with the (lack of) compliance with preventive measures. Nevertheless, little is known about their stability over time and across behaviors. As a result, using a longitudinal approach, the present paper aims at extending the findings of Wollast, et al. [5] and related studies by selecting a greater set of predictors considered simultaneously, aiming to offer a more comprehensive picture of health behavior adherence over time.

### National context

**Fig 1:**
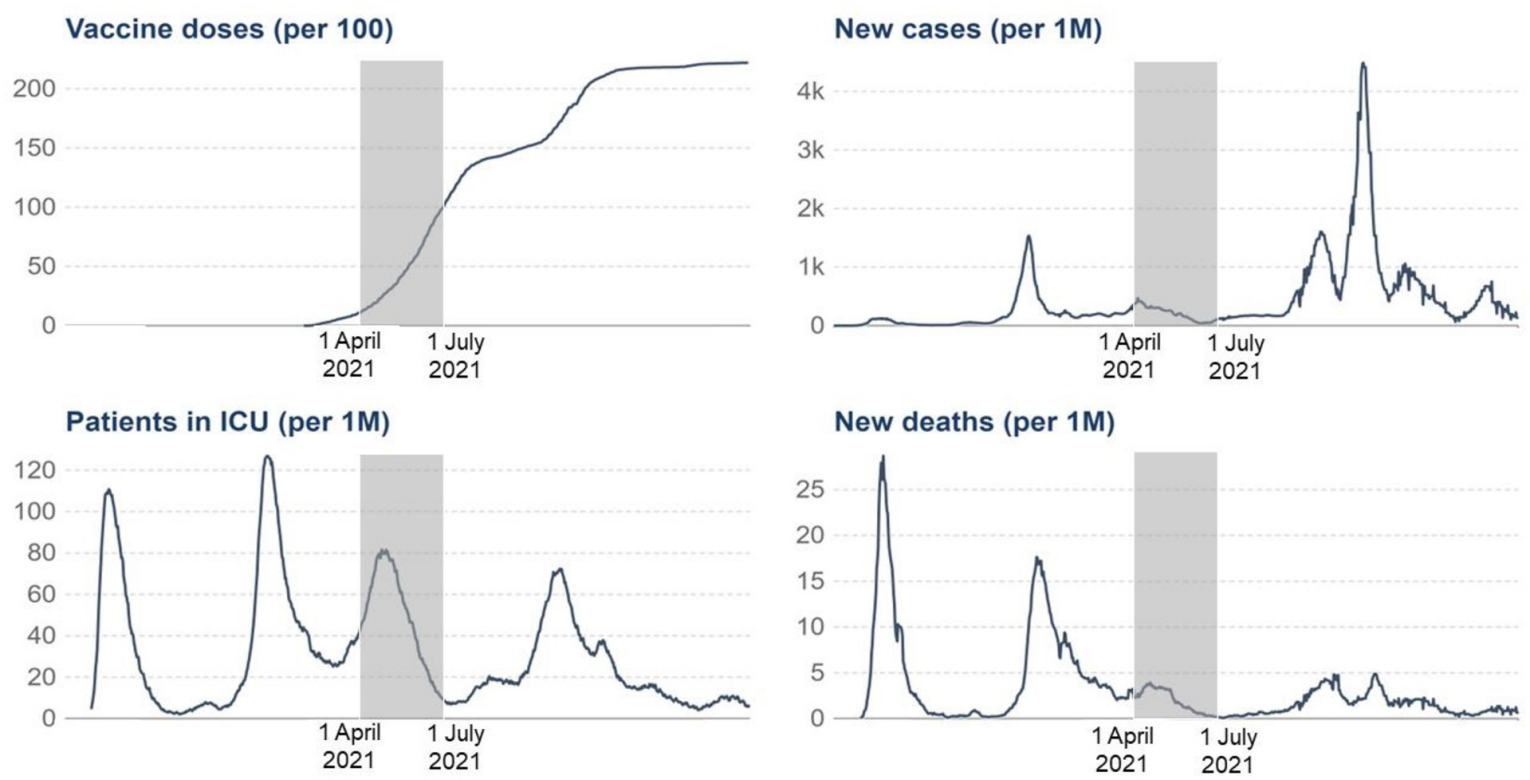
Vaccine doses, daily new confirmed cases, ICU patients, and death cases in Belgium. The gray area indicates the period of data collection (adapted from Mathieu et al., 2023).

**Fig 2:**
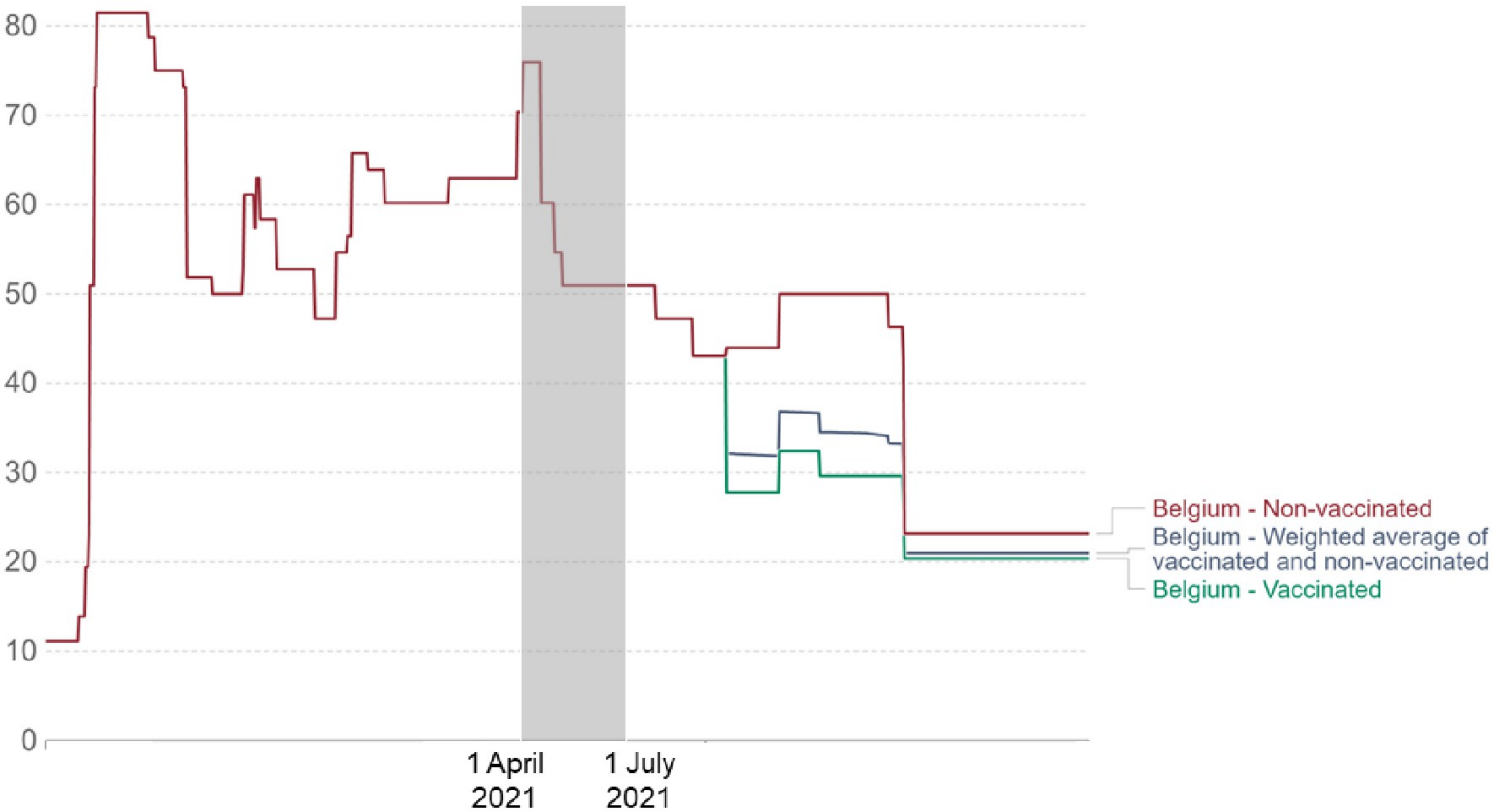
Stringency index in Belgium. The stringency index is a composite measure based on nine response indicators, including school closures, workplace closures, and travel bans rescaled to a value from 0 to 100 (100 = strictest). If policies vary at the subnational level, the index shows the response level of the strictest subregion. The gray area indicates the period of data collection (adapted from Mathieu et al., 2023).

Data for the present study were collected between April 1st and July 2nd, 2021, in Belgium, coinciding with the third wave of COVID-19. Fig 1 shows data on vaccine doses, COVID-19 cases per million people, ICU patients, and mortality cases, while Fig 2 illustrates the stringency of measures in Belgium [41]. Initially, the numbers were high, reflecting the gravity of the situation, but they substantially decreased as we reached our latest data collection points, indicating a less critical epidemiological condition. Lockdown policies, including mandatory mask wearing, social contact limitations, and 1.5-meter (i.e., five feet) physical distancing, were in place during the severe phase, with handwashing strongly recommended. As the epidemiological situation improved, these restrictions eased, aligning with our latest data collection phase. In summary, our data collection period offers a unique opportunity to study the predictors of health behavior adherence in a context that shifted from a severe health crisis and strict measures to a less critical situation with more relaxed measures.

### The present research

The review above highlights a prevailing trend in COVID-related studies, which predominantly employed one-time-point designs and examined only a restricted set of predictors simultaneously. Only a handful of studies [5, 22] utilized longitudinal designs and considered a broader spectrum of predictors. Another significant limitation found in the literature is the assessment of health behaviors through a single or very limited number of items, potentially compromising measurement quality and ecological validity. The present study addressed these limitations by investigating the predictors of four primary health behaviors (handwashing, mask wearing, social contact limitations, and physical distancing) while mitigating these measurement-related issues.

## Methods

The present study complies with the APA ethical regulations for research on human subjects, and all participants gave online written informed consent, as approved by the institutional review boards of the principal investigator (Project 2021–13). Raw data, syntax, items, and additional elements can be found in the online supplementary material (https://osf.io/zyc42/).

### Participants and procedure

A total of 5803 French-speaking participants from Belgium were involved in the current longitudinal study. Table 1 provides information on the number of participants and their characteristics for each of the five data collection time points. Data collection occurred over five waves spanning April 1 to July 2, 2021, with approximately three weeks between each wave and each wave lasting 7–8 days. This period coincided with the third wave of the pandemic and the rollout of intensive vaccination campaigns (Fig 1).

**Table 1:** Descriptives and comparisons across time for the study variables.

	Range	T1	T2	T3	T4	T5
Health behaviors						
Handwashing	1–5	3.54^A^	3.47^AB^	3.47^AB^	3.42^BC^	3.33^C^
Mask wearing	1–5	3.76^A^	3.76^A^	3.76^A^	3.62^B^	3.37^C^
Social contact limitations	1–5	3.63^A^	3.59^AB^	3.53^B^	3.38^C^	3.15^D^
Physical distancing	1–5	3.62^A^	3.58^AB^	3.52^B^	3.34^C^	3.14^D^
Demographics						
Age	18–92	51.07^A^	52.77^B^	53.34^BC^	53.74^BC^	54.43^BC^
Gender (female)	0–1	57%^A^	60%^AC^	62%^BC^	63%^BC^	62%^BC^
University (yes)	0–1	78%^A^	82%^B^	84%^B^	85%^B^	85%^B^
Emotions						
Upset	1–5	2.95^A^	2.69^B^	2.46^C^	2.33^D^	2.19^E^
Lively	1–5	2.88^A^	2.97^A^	3.08^A^	3.16^AB^	3.18^B^
Empathetic	1–5	3.49^A^	3.49^A^	3.50^A^	3.48^A^	3.51^A^
Fearful	1–5	2.03^A^	1.92^B^	1.80^C^	1.74^CD^	1.68^DE^
CCCM						
Intention	1–5	3.92^A^	3.89^A^	3.93^A^	3.79^B^	3.71^B^
Attitude	1–5	3.97^AB^	3.98^AB^	4.05^A^	3.95^BC^	3.89^C^
Perceived control	1–5	3.29^A^	3.28^A^	3.33^A^	3.25^A^	3.24^A^
Habit	1–5	3.35^A^	3.33^A^	3.35^A^	3.27^AB^	3.22^B^
Norms	1–5	3.73^A^	3.71^AC^	3.75^A^	3.64^BC^	3.56^B^
Risks	1–5	3.37^A^	3.29^B^	3.21^C^	3.00^D^	2.88^E^
Motivation						
Autonomous	1–5	3.93^AB^	3.91^ABC^	3.98^A^	3.87^B^	3.83^BC^
Controlled	1–5	3.32^A^	3.32^A^	3.31^A^	3.25^A^	3.13^B^
Covid-related						
Infection status	-2 to 2	-0.44^A^	-0.48^A^	-0.51^A^	-0.49^A^	-0.52^A^
Vaccinated (yes)	0–1	24%^A^	45%^B^	69%^C^	79%^D^	89%^E^

*Note*: CCCM = Central components of socio-cognitive models of behavioral change; Means are provided for continuous variables and percentages (representing the level in parenthesis) for binary variables. Values from the same row with different superscripts are statistically different (*p* < .05). Multiple comparison tests were adjusted using the Bonferroni method.

The participants were invited to complete an online survey distributed via mailing lists, social media platforms, and news outlets during the first two time points (T1 and T2). The survey’s initial section included our predictors and outcome variables, followed by requests for socio-demographic information. Upon completing the questionnaire, the participants received a debriefing and were given the option to express their willingness to be contacted for follow-up data collection. Additionally, they had the opportunity to enter a gift lottery after each wave (for more information about the data collection and sample characteristics, see [5]).

### Missing values and dropout rates

Given the longitudinal nature of the present work, we provide information regarding missing values and dropout rates. S1 Fig (available in the online supplementary) reports the total number of waves of data collection completed per participant. S2 Fig reports the attrition rate for the full sample.

### Measures

#### Health behaviors

Adherence to the four health behaviors was evaluated using seven items for each behavior rated on a response scale ranging from “1 = Almost never” to “5 = Very often.” These items have undergone validation by Wollast and colleagues [5] and encompass a wide range of social situations. They were meticulously crafted to align with the prevailing measures in effect during COVID-19, accounting for context sensitivity (e.g., private vs. public settings) and varying degrees of application difficulty (e.g., interactions with strangers vs. relatives). Illustrative items include the following: “I wash/disinfect my hands before and after entering and leaving a public space” (handwashing), “I wear a mask when I am with friends who are not part of my close contacts” (mask wearing), “I engage in social interactions with people who are not part of my household” (social contact limitations), and “I maintain a physical distance from people who are not part of my household” (physical distancing). All measures consistently demonstrated strong internal consistency at all time points, with all McDonald’s omega coefficients (ω) exceeding .89.

#### Central components of behavioral change

We assessed the central components of socio-cognitive models of behavioral change for each of the four health behaviors by using a single item for each component on a 5-point Likert scale ranging from “1 = Totally disagree” to “5 = Totally agree,” except in the case of the *norms*, for which we relied on three items.

The items were the following: “I am willing to [health behavior] as often as possible” (intentions), “I am confident that [health behavior] will limit the spread of COVID-19” (attitudes), “For me, [health behavior] is easy” (perceived control), “[Health behavior] is something I do without thinking” (habit), “People who are important to me follow [health behavior] procedures” (descriptive/subjective norms), “Most people who are important to me think I should follow [health behavior]” (prescriptive norms), and “It is the right thing to follow [health behavior] procedures” (moral norms). The three normative components were averaged into a single component (labeled “Norms”) to reduce the number of predictors and because of their very high correlations (norms’ McDonald’s omega coefficients ≥ .89). Perceived risk of infection (based on [19]) was assessed with the following item: “In your opinion, what is your risk of becoming infected with COVID-19 in the near future if you do not follow the [health behavior] measure?” (from 1 = Very small to 5 = Very high).

#### Emotions

The participants indicated their current emotional states based on the French version of the Positive and Negative Affect Scale–State (PANAS [42, 43]) on a scale ranging from “1 = Totally disagree” to “5 = Totally agree.” A principal component analysis revealed a four-dimensional structure (analysis available in the online repository). The “upset” component (all ω*s* ≥ .84) comprised the emotional states of annoyed, irritable, angry, agitated, and sad. The “lively” component (all ω*s* ≥ .79) encompassed the emotional states of strong, decided, active, enthusiastic, exhilarated, and proud. The “empathetic” component (all ω*s* ≥ .63) encompassed the emotional states of attentive, careful, and compassionate. The “fearful” component (all ω*s* ≥ .80) comprised the emotional states of distressed, guilty, scared, afraid, anxious, and surprised. Note that these four dimensions successfully replicated those found in the literature [7].

#### Motivation

The autonomous and controlled motivation to comply with the health behaviors [9] were assessed by a single item each for each behavior, with response options ranging from “1 = Totally disagree” to “5 = Totally agree.” Autonomous motivation was assessed with “During the last 7 days I followed [health behavior] because I personally think that it is meaningful” and controlled motivation with “During the last 7 days I followed [health behavior] because I felt obligated to do so.”

#### COVID-related measures

The participants were asked if they were infected (2 = tested positive, 1 = probably positive, -1 = probably negative, -2 = tested negative) or if they had received at least one dose of the vaccine (yes or no).

#### Demographics

The participants provided information about their age, gender, and level of education.

### Statistical analyses

We first present descriptive statistics and conduct mean or proportion comparisons across different time points for our study variables. To provide a complementary approach to the group-based trajectory modeling used in Wollast and colleagues [5], we employed separate multiple linear regression models for each time point and each behavior.

## Results

### Descriptives of the study variables

Table 1 presents descriptive statistics for all the study variables at each data collection time point. Adherence to the four health behaviors remained relatively stable from T1 to T3, with a decrease observed in T4 and T5, especially for avoidant behaviors, which is in line with the epidemiological situation in Belgium (see Fig 1). Regarding demographics, the sample composition across waves remained notably consistent in terms of age, education, and gender. In terms of emotions, there was a consistent decline in the scores for the “upset” and “fearful” factors over time, while the “lively” factor exhibited an increase. The “empathetic” factor remained relatively stable, with no significant change observed. Turning to the CCCM, the trend mirrored the stability observed in health behavior adherence, with relative stability from T1 to T3 and a slight decrease in T4 and T5. Regarding COVID-related measures, infection status remained steady over time, while the vaccination rate demonstrated a rapid increase. In summary, these fluctuations in health behavior adherence, emotional states, and behavioral components aligned perfectly with the decrease in social distancing rules because of the improvement of the general sanitary situation in Belgium.

### Health behavior predictions

Tables 2–5 provide the regression model estimates for each health behavior at each time point. All the regression models achieved remarkable levels of explained variance (all adjusted *R*^*2*^ *>* .60). Below, we describe the general and consistent trends for each behavior.

**Table 2:** Predictors of handwashing across the five waves.

	Unstandardized beta coefficient (B)				
	T1	T2	T3	T4	T5
Constant	-0.386***	-0.477**	-0.522**	-0.269	-0.402*
Age	-0.004***	-0.001	-0.005***	-0.005***	-0.004***
Gender (female)	0.159***	0.136***	0.174***	0.152***	0.124***
University (yes)	0.020	0.107***	0.055	0.019	-0.026
Emotions: upset	-0.020	-0.010	-0.009	-0.013	-0.035
Emotions: lively	-0.017	-0.025	0.026	-0.026	-0.004
Emotions: empathetic	0.090***	0.059**	0.067***	0.059*	0.039
Emotions: fearful	0.082***	0.079***	0.125***	0.098**	0.142***
Intention	0.216***	0.223***	0.234***	0.205***	0.169***
Attitude	-0.004	0.045*	0.055*	0.089***	0.126***
Perceived control	0.097***	0.067***	0.114***	0.130***	0.131***
Habit	0.162***	0.194***	0.144***	0.129***	0.157***
Norms	0.157***	0.165***	0.121***	0.072*	0.068*
Risk	0.176***	0.183***	0.194***	0.194***	0.172***
Autonomous	0.119***	0.059*	0.039	0.083**	0.096**
Controlled	0.002	-0.002	0.003	0.000	0.017
Have COVID-19 (yes)	-0.032***	-0.028*	-0.044***	-0.035*	-0.028
Vaccinated (yes)	0.021	-0.010	0.162***	0.176***	0.120*
Model’s adjusted R^2^	.628***	.646***	.636***	.651***	.648***

*Note*: Significant effects (*p* < .05) are in bold.

*p < .05.

**p < .01.

***p < .001.

**Table 3:** Predictors of mask wearing across the five waves.

	Unstandardized beta coefficient (B)				
	T1	T2	T3	T4	T5
Constant	0.052	-0.067	-0.131	0.477*	-0.223
Age	0.005***	0.007***	0.003*	0.003*	0.004**
Gender (female)	0.128***	0.096***	0.076*	0.040	0.025
University (yes)	0.108***	0.099***	0.053	0.027	0.023
Emotions: upset	-0.034*	-0.001	0.035	-0.009	0.072*
Emotions: lively	-0.079***	-0.082***	-0.046	-0.114***	-0.012
Emotions: empathetic	0.093***	0.057**	0.071**	0.033	0.027
Emotions: fearful	0.062***	0.039	-0.006	0.028	0.039
Intention	0.209***	0.182***	0.285***	0.176***	0.180***
Attitude	0.004	0.075**	0.003	0.116***	0.067
Perceived control	0.029*	0.031*	0.028	0.028	0.034
Habit	0.082***	0.065***	0.064***	0.075***	0.114***
Norms	0.238***	0.218***	0.192***	0.193***	0.219***
Risk	0.123***	0.137***	0.130***	0.125***	0.165***
Autonomous	0.161***	0.177***	0.191***	0.147***	0.114***
Controlled	-0.025**	-0.028**	-0.019	-0.026	-0.040**
Have COVID-19 (yes)	-0.030**	-0.007	-0.037**	-0.002	-0.035*
Vaccinated (yes)	-0.043	-0.031	0.086*	0.062	0.025
Model’s R^2^	.645***	.654***	.674***	.644***	.649***

*Note*: Significant effects (*p* < .05) are in bold.

*p < .05.

**p < .01.

***p < .001.

**Table 4:** Predictors of social contact limitations across the five waves.

	Unstandardized beta coefficient (B)				
	T1	T2	T3	T4	T5
Constant	0.842***	1.026***	0.680***	0.780***	0.702***
Age	0.006***	0.006***	0.005***	0.006***	0.006***
Gender (female)	0.125***	0.125***	0.121***	0.052	0.005
University (yes)	-0.009	-0.060	-0.039	-0.052	-0.068
Emotions: upset	0.002	-0.013	0.021	-0.007	0.024
Emotions: lively	-0.094***	-0.129***	-0.067**	-0.105***	-0.104***
Emotions: empathetic	0.057***	0.056**	0.036	0.009	0.008
Emotions: fearful	0.026	-0.003	0.022	0.075*	0.045
Intention	0.216***	0.225***	0.216***	0.231***	0.251***
Attitude	0.018	0.034*	0.003	0.019	0.018
Perceived control	0.003	0.001	-0.019	0.019	0.008
Habit	0.071***	0.072***	0.093***	0.092***	0.094***
Norms	0.169***	0.154***	0.158***	0.130***	0.131***
Risk	0.069***	0.072***	0.060***	0.080***	0.084***
Autonomous	0.114***	0.121***	0.174***	0.133***	0.123***
Controlled	0.056***	0.046***	0.039***	0.042***	0.023
Have COVID-19 (yes)	-0.034***	-0.017	-0.022	-0.036**	-0.029*
Vaccinated (yes)	-0.066**	-0.025	0.036	0.065	0.015
Model’s R^2^	.652***	.651***	.651***	.657***	.662***

*Note*: Significant effects (*p* < .05) are in bold.

*p < .05.

**p < .01.

***p < .001.

**Table 5:** Predictors of physical distancing across the five waves.

	Unstandardized beta coefficient (B)				
	T1	T2	T3	T4	T5
Constant	0.640***	0.565***	0.489**	0.480**	0.361
Age	0.001	0.001	0.001	0.001	0.001
Gender (female)	0.128***	0.147***	0.107***	0.075*	0.056
University (yes)	-0.099***	-0.096**	-0.124**	-0.115*	-0.125*
Emotions: upset	-0.049***	-0.053**	-0.012	-0.013	0.008
Emotions: lively	-0.120***	-0.132***	-0.154***	-0.136***	-0.154***
Emotions: empathetic	0.073***	0.069***	0.067**	0.008	0.024
Emotions: fearful	0.059***	0.074***	0.043	0.113***	0.097*
Intention	0.269***	0.237***	0.224***	0.209***	0.238***
Attitude	-0.030*	0.038	0.049	-0.001	0.007
Perceived control	0.008	0.053***	0.036	0.083***	0.100***
Habit	0.083***	0.072***	0.095***	0.090***	0.077**
Norms	0.178***	0.141***	0.227***	0.250***	0.222***
Risk	0.086***	0.096***	0.124***	0.103***	0.119***
Autonomous	0.186***	0.178***	0.098***	0.118***	0.100**
Controlled	0.036***	0.032***	0.015	0.022	0.018
Have COVID-19 (yes)	-0.027**	-0.033**	-0.026	-0.038*	-0.030
Vaccinated (yes)	-0.058*	-0.043	0.022	0.033	-0.041
Model’s R^2^	.639***	.649***	.641***	.633***	.642***

*Note*: Significant effects (*p* < .05) are in bold.

*p < .05.

**p < .01.

***p < .001.

Handwashing (see Table 2) was positively predicted by gender (female), empathetic, and fearful emotional factors, the central components of behavioral change, autonomous motivation, and perceived risks of infection. Age had a negative, although small, influence. Taken individually, age showed a positive association with handwashing, suggesting that older individuals washed their hands more frequently than their younger counterparts. However, in the regression model, when accounting for other factors, a negative coefficient emerged. It is important to note that in the regression model (Bs = -.004***), as well as in the correlations (rs > .04***), coefficients were small and negligible. Importantly, the majority of predictors remained significant across all five waves of data collection, except for vaccine intention, which only became significant at T3, T4, and T5.

Mask wearing (see Table 3) was positively predicted by age (older people), gender (female), and education (went to the university). It is worth noting that the latter two demographic factors lost statistical significance in the concluding waves of data collection. Moreover, mask wearing was positively associated with fearful and empathetic emotional factors, mainly at the beginning of the data collection. Conversely, a negative association was observed between lively emotional factors and mask wearing. Furthermore, mask wearing was consistently and positively predicted by the central components of behavioral change, although with less consistent associations for attitude and perceived control. Noteworthy is the positive influence of autonomous motivation on mask wearing, while controlled motivation demonstrated a negative association with this behavior.

Social contact limitations (see Table 4) were positively predicted by gender (female) until T3, age (older individuals), the empathetic factors until T2, the central components of behavioral change (except for attitude and perceived control), and both autonomous and controlled motivation. Conversely, it was negatively and consistently predicted by the lively emotional factor.

Physical distancing (see Table 5) was positively associated with gender (female), fearful, and empathetic emotional factors primarily at the onset of data collection, the central components of behavioral change (excluding attitude), autonomous motivation, and controlled motivation, the latter of which only during T1 and T2. Conversely, the lively emotional factor and university attendance negatively and consistently predicted adherence to physical distancing.

Overall, women were more likely to adhere to health behaviors than men. Furthermore, the lively emotional factor had a negative impact on avoidant behaviors (mask wearing, social contact limitations, and physical distancing), whereas it was the opposite for the empathetic component. In the same vein, fear positively predicts adherence to health behaviors. Furthermore, the central components of behavioral change—intention, habit, and norms—were consistently important predictors across all behaviors. Autonomous motivation emerged as another major predictor across behaviors over time, whereas controlled motivation had inconsistent or negative effects on health behavior adherence. Finally, the perceived risks associated with not complying with each health behavior positively impacted adherence levels across all measurement points.

## Discussion

Highlighting the factors that either promote or hinder adherence to health behaviors is crucial for preparing for future health crises. Although there is a wealth of research in this area, much of it suffers from several limitations, such as relying on one-time-point designs, including a limited number of predictors, and using single-item-based outcomes. The present research overcame these limitations by employing a five-time-point longitudinal design, incorporating a wider range of emotional, cognitive, and behavioral predictors and utilizing multi-item health behavior variables. Importantly, this longitudinal study addressed these limitations by utilizing a large sample comprising thousands of participants, thereby ensuring substantial statistical power to draw solid conclusions.

Our findings indicate a consistent decrease in adherence levels over time, particularly notable during the T4–T5 time points. Interestingly, handwashing, a relatively straightforward and traditional behavior, demonstrated a slower decline compared with the three other health behaviors. Notably, health behaviors involving avoidant measures, such as wearing a mask, limiting social contact, and practicing physical distancing, demonstrated a greater decline, especially during the last two waves. These results align with previous research suggesting that behaviors requiring avoidance or social restraint may be more challenging to sustain over time because of associated psychological costs [4, 5]. Specifically, Wollast et al. [5] found that those participants with high levels of adherence to social contact limitations and physical distancing experienced greater loneliness and lower life satisfaction over time (note that this effect was marginal for mask wearing and nonsignificant for handwashing). Despite fluctuations in adherence levels, there remained a noticeable commitment to these sanitary measures throughout the study. This underscores the ongoing importance of public health efforts aimed at encouraging and supporting individuals to adopt and sustain these behaviors.

A similar trend was observed for the central components of behavior change, including intention, attitude, control, habit, norms, and risk, as well as motivation. Emotions associated with upset (i.e., annoyed, irritable) and fear (i.e., scared, afraid) emotional factors also followed a comparable pattern. Similarly, the trend was opposite for the lively emotional factor, which included being active and enthusiastic. This pattern of behaviors, emotions, attitudes, and motivation clearly reflects an adaptive response to the epidemiological situation during our data collection period, as depicted in Figs 1 and 2. Initially, during the third pandemic wave (T1–T3), we observed high levels of infections, hospitalizations, and deaths, accompanied by stringent measures. However, as the number of COVID-19 cases and hospitalization burden decreased, and pandemic-related measures were relaxed (T4–T5), the situation became less critical.

Significantly, the empathetic emotional factor, including qualities such as attentiveness and compassion, demonstrated stability throughout this time frame. This implies that people’s concern for others was activated and consistent over time. This outcome contradicts earlier research that documented a detrimental impact on empathic social skills resulting from the pandemic outbreak [44].

### Predictors of health behaviors

In terms of prediction, our results indicate that most of the explanatory variables considered in the present study demonstrated significant associations with health behaviors (above and beyond other predictors). Importantly, all the regression models achieved remarkable levels of explained variance, which indicates that the set of selected predictors is highly effective in capturing and accounting for the variability in health behavior adherence.

Consistent with previous findings [6–8], we observed that women were more likely to adhere to health behaviors, while the effects of age and education were either inconsistent or negligible. Regarding emotions, the empathetic factors had a positive effect on the avoidant behaviors (mask wearing, social contact limitations, and physical distancing), whereas it was the opposite for the lively factor. This suggests that caring about others helps people adhere and stick to more difficult and costly measures because it provides a means to protect others from the virus. This is consistent with findings showing that individuals with greater empathic concern tend to engage in more preventive health behavior over time [45–47]. Also, it seems logical that those who feel livelier—and, thus, are likely to also be more sociable—are less prone to comply with preventive behaviors that run counter to socialization [48]. Finally, it is worth mentioning that fear also plays a smaller but significant role in promoting adherence to health behaviors. This suggests that fear of potential consequences or government sanctions may serve as a partial motivator for adherence.

Regarding motivation, the results revealed that autonomous motivation, as opposed to controlled motivation, emerged as a robust and enduring predictor of all health behaviors, consistent with previous research [9] and SDT [38]. This highlights the significance of distinguishing between different motivational types qualitatively rather than solely relying on quantitative assessments (i.e., high versus low overall motivation). Indeed, autonomous motivation tends to yield positive and lasting effects because it originates from within the individual when they have internalized and taken ownership of the measures, finding them meaningful, coherent, and aligned with their personal values. In contrast, controlled motivation often has a shorter duration because adherence tends to cease once external pressures are removed. However, controlled motivation displayed weaker but consistent and positive associations with social contact limitations and physical distancing. Given that these behaviors are among the most challenging to practice, they may require ongoing external motivation for individuals to adhere to them over extended periods. However, excessive external pressure can have adverse effects, leading to reactance or defiance, as we observed for a negative association with mask wearing [40].

The central components of socio-cognitive models of behavioral change had the strongest positive and a prolonged impact on health behaviors. This is consistent with previous findings [3, 7, 28, 29] and provides further support for the TPB [25, 49] and, to some extent, the HAPA models [26] in a long-lasting pandemic context. Among these predictors, risk perception of being infected because of not following the measures had a large effect on levels of adherence (see also [32]), suggesting that these preventive behaviors are indeed perceived as an effective tool to combat infection. Interestingly, however, more objective indicators of risk, such as infection status or vaccination, had a minimal and inconsistent impact, thus emphasizing the key role of the subjective perception of risks in explaining adherence to protective behaviors.

### Implications and future directions

The research on health behaviors is flourishing, and the associations found in the present study significantly contribute to the literature, offering implications for both research and practice. Many studies focusing on health behaviors have adopted a cross-sectional approach, relying on single-item measures for each sanitary behavior and often neglecting comprehensive sets of predictors. The current study has addressed these limitations by (1) employing a longitudinal design spanning five waves during the COVID-19 pandemic, (2) utilizing validated measures for health behaviors, (3) exploring a broad and diverse array of predictors, and (4) presenting positive and encouraging outcomes that can inform and support public health professionals and policymakers.

The present study has crucial practical implications for public health professionals and policymakers. The dynamic interplay of factors influencing health behaviors over time suggests the need for personalized and adaptive interventions. Public health campaigns can refine communication strategies, emphasizing the positive impact of health behaviors and collective responsibility in mitigating health crises (see [32]). These campaigns could also emphasize the individual’s role and responsibility in overcoming the crisis to foster autonomous and intrinsic motivation [40]. In addition, effective communication strategies that promote emotions of empathy, prosocial motives, and unity within the population can enhance individuals’ commitment to health behaviors [50]. This could be achieved by acknowledging the importance of adhering to social distancing rules, recognizing the challenges people face, and conveying a sense of collective solidarity, such as the message that “we are all in this together” [51, 52]. Regarding risk perception, we recommend avoiding anxiety-inducing communication, such as repetitive negative information. Instead, it is advisable to provide concrete and objective information about the risks of infections and potential consequences of illnesses [32]. Importantly, policymakers should use an autonomy-supportive communication style by being empathic, highlighting the relevance and necessity of (stringent) measures, treating citizens as responsible and benevolent agents, and using noncontrolling language to preserve the population’s volitional commitment to policy-based measures (for additional recommendations, see [39]).

In summary, the present study not only advances theoretical understanding, but also provides actionable insights for health professionals and public health practitioners. The nuanced exploration of the factors influencing health behaviors over time calls for a paradigm shift in interventions, emphasizing adaptability, empathy, and intrinsic motivation for more targeted and impactful strategies in future health crises.

### Limitations

Despite its theoretical and practical implications, the current work is not without its limitations. In particular, the results may be applicable only to populations with similar sample characteristics, specifically Belgian residents who are educated and middle-aged. Future research should aim to expand and replicate our findings in more diverse and broader populations [20, 53, 54]. Another limitation is that we did not encompass all the health behaviors recommended by health authorities. For instance, more recent guidelines have emphasized the importance of ventilation as a key measure to prevent the spread of the virus. Additionally, we acknowledge that our study did not incorporate every component of the HAPA model, such as self-efficacy, outcome expectancies, planning, action planning, and coping [26]. This omission was because of the length constraints of our survey. Therefore, we encourage future research to include these elements in their models because they may provide additional predictive value. Finally, our methodology entails the use of individual regression models for each time point of data collection rather than adopting a simultaneous analysis across all times, as in multilevel or trajectory analysis (see [55]). This method, for instance, does not allow for a precise statistical exploration of the stability and changes in predictors over time. This deliberate approach was chosen to extend and replicate certain aspects of Wollast et al. [5] findings. Although these researchers employed group-based trajectory modeling, our intentional decision to conduct separate analyses gave an opportunity to provide complementary perspectives into the nuanced relationship between predictors and health behaviors over extended periods. This approach has allowed for a more precise examination of each individual predictor’s impact at each time point.

In conclusion, our longitudinal investigation into health behaviors during the COVID-19 pandemic revealed the robust dynamics of several predictors influencing individual responses, providing valuable recommendations for policymakers and public health authorities. The decreasing adherence levels to health behaviors underscore the challenge for the population to sustain these rules over time. In this context, we suggest employing effective and mindful strategies to encourage people to adopt sanitary behaviors during future crises. For instance, the enduring impact of empathetic factors and autonomous motivation emphasizes the potential to nurture the intrinsic drivers of health behavior adherence. As we anticipate future health crises, the lessons learned during COVID-19 advocate for a paradigm shift toward evidence-based health interventions finely tuned to strike a balance between physical and mental health.

## Supporting information

S1 Fig Total number of waves completed. *Note*. The figure shows the number of waves that respondents completed out of a total possible five waves (e.g., 772 participants completed five waves).
(PDF)

S2 Fig (PNG)
