# Supplementary material for: Predicting health behaviors during the COVID-19 pandemic: A longitudinal study
Source: PLoS One. 2024 Mar 15;19(3):e0299868. doi: 10.1371/journal.pone.0299868 (PMC10942062; doi:10.1371/journal.pone.0299868)
Supplement: S1 Fig — Note. The figure shows the number of waves that respondents completed out of a total possible five waves (e.g., 772 participants completed five waves). (PDF) [file pone.0299868.s001.pdf]

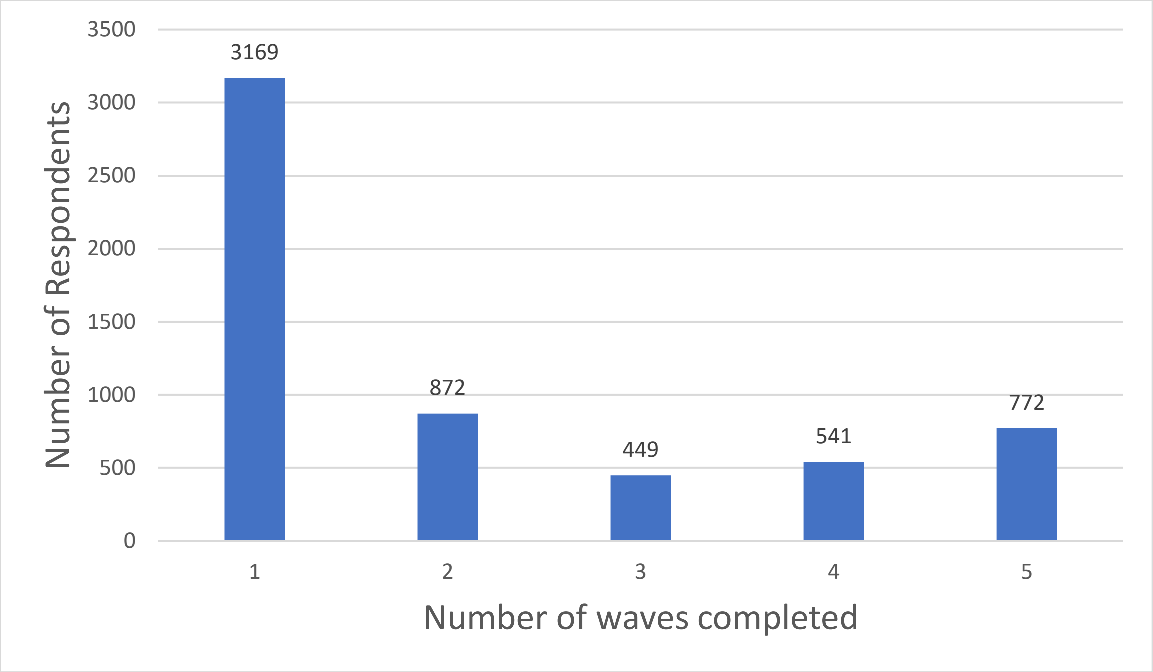

Figure S1. *Total number of waves completed.*

Note. The figure shows the number of waves that respondents completed out of a total possible five waves (e.g., 772 participants completed five waves).
