# Supplementary figures and images for: Predicting health behaviors during the COVID-19 pandemic: A longitudinal study
Source: PLoS One. 2024 Mar 15;19(3):e0299868. doi: 10.1371/journal.pone.0299868 (PMC10942062; doi:10.1371/journal.pone.0299868)

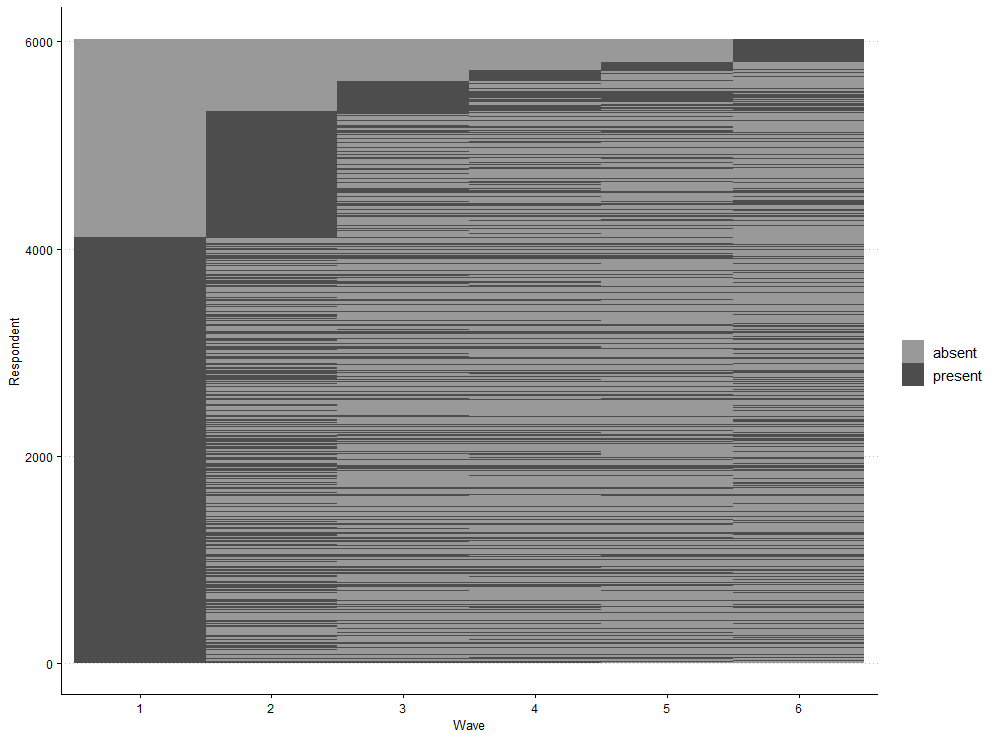

Supplement: S2 Fig — (PNG) [file pone.0299868.s002.png]
